# Antibiotic Exposure in School Children in Tropical Environments: Impact of Dietary Habits and Potential Health Risks

**DOI:** 10.3390/toxics13121089

**Published:** 2025-12-18

**Authors:** Lin Zhao, Xin-Yu Wang, Yang Xiang, Ting-Ting Xu, Shi-Jian Liu, Xiao-Ya Lin, Ying Guo

**Affiliations:** 1 Guangdong Key Laboratory of Environmental Pollution and Health, School of Environment and Climate, Jinan University, Guangzhou 511443, China; zl1013@stu.jnu.edu.cn (L.Z.); xinyuwang@stu2023.jnu.edu.cn (X.-Y.W.); 880216bkpp@stu2023.jnu.edu.cn (Y.X.); xutingting@jnu.edu.cn (T.-T.X.); linxiaoya@stu2025.jnu.edu.cn (X.-Y.L.); 2 Clinical Research Unit, Shanghai Municipal Hospital of Traditional Chinese Medicine, Shanghai University of Traditional Chinese Medicine, Shanghai 201203, China; 3 Hainan Branch, Shanghai Children’s Medical Center, School of Medicine, Shanghai Jiao Tong University, Sanya 572000, China

**Keywords:** antibiotics, dietary habits, exposure health assessment, oxidative stress, MDA, 8-OHdG

## Abstract

Due to their wide application, there is a large amount of residual antibiotics in our environment and food, raising concerns about health risks to children. In this study, 302 primary-school students in Hainan Province, China, were recruited to collect urine samples and questionnaires. The internal exposure levels of sixteen antibiotics and three metabolites in urine were determined by high-performance liquid chromatography–tandem mass spectrometry (HPLC-MS/MS), and the contents of DNA oxidative damage markers, 8-hydroxy-2′-deoxyguanosine (8-OHdG) and lipid peroxidation marker malondialdehyde (MDA), were also measured. Antibiotics and their metabolites were frequently detected, with a total concentration of < LOD-4.58 × 10^3^ ng/mL. Binary logistic regression analysis revealed that the detection frequency of DFs of antibiotics was associated with animal-derived foods, such as red meat with fluoroquinolones (FQs) (OR = 76.4, 95% CI 1.68–3479), poultry with norfloxacin (NFX) (OR = 6.56, 95% CI 1.08–39.9), and aquatic products with ciprofloxacin (CIP) (OR = 3.96, 95% CI 1.32–11.9). Cumulative risk assessment based on microbial effects showed a hazard index of 3.5 for children, mainly driven by azithromycin (45.6%), oxytetracycline (18.1%), and CIP (33.9%). Multiple linear regression indicated that lipid peroxidation was significantly associated with high quantiles of three antibiotic classes, while DNA oxidation was positively correlated with all antibiotic classes except FQs. These findings indicate that children in Hainan are widely exposed to antibiotics. Although the exposure levels are generally low, chronic low-dose antibiotic exposure may contribute to disease development and oxidative stress damage.

## 1. Introduction

In 1947, S. A. Waksman defined an antibiotic as “a chemical substance produced by microorganisms that has the ability to inhibit or even destroy the growth of bacteria and other microorganisms”, and today, an antibiotic refers to a natural or man-made antimicrobial substance [1]. The clinical use of antibiotics is the greatest medical breakthrough of the 20th century. Although antibiotics revolutionized the treatment of infectious diseases across the globe, antibiotic resistance has also become a major threat. For example, methicillin-resistant Staphylococcus aureus (MRSA) mutates a methicillin-resistant structural gene (the mecA gene or SCCmec gene) to inhibit the development of methicillin-resistant bacteria [2]. MRSA caused more than 100,000 infections in 2019 [3]. It is predicted that antibacterial resistance may lead to 10 million deaths by 2050.

Antibiotics are used not only in human medicine, but also in veterinary medicine. Unused and unmetabolized antibiotics may enter the aquatic environment through wastewater. If they are not degraded or eliminated, they may enter surface water and groundwater, and even drinking water. In addition, veterinary antibiotics will be excreted by animals into manure, which is used as agricultural fertilizer, and may also enter groundwater into the soil [4]. At present, antibiotic residues have been detected in various environmental media such as surface water, sediments, agricultural soils, fish species, and vegetables [5]. As a result, residues of multiple antibiotics have been found in the meat, egg products, milk, and vegetables on our dinner tables [6,7].

In recent years, the residues of antibiotics in food have attracted more and more attention. It is reported that antibiotics can destroy the balance of the human intestinal microbial symbiotic population, leading to the occurrence of potential inflammation and increasing the probability of autoimmune diseases [8,9]. Long-term exposure to antibiotics has been shown to be associated with an increased risk of type 2 diabetes, independent of traditional diabetes risk factors [10,11]. In addition, antibiotics are also associated with a range of health problems, such as obesity, colorectal adenoma, cardiovascular mortality, type 1 diabetes, multiple sclerosis, and Crohn’s disease [12,13,14,15], which are related to the intestinal microbiota. In addition, fluoroquinolones may carry the risk of cartilage damage and irreversible cartilage toxicity, which may lead to permanent claudication. They should be used on children with caution [16]. Chloramphenicol and its derivatives may be at risk of severe bone marrow suppression, including specific aplastic anemia [17].

Because neither intestinal flora nor the immune system are fully developed in children, they are more susceptible to antibiotics than adults. In addition, in the case of low doses, the impact of antibiotics on children is also more obvious [18]. A number of studies have shown that long-term exposure to low-dose antibiotics is associated with adverse health effects in children, such as obesity, asthma, allergic rhinitis, food allergy, mood disorders, and anxiety disorders [19,20,21,22,23]. Antibiotic exposure in children has been reported in several cities in different climate zones. For example, in China, the related data were from Shandong Province with a temperate monsoon climate, Shanghai, Jiangsu Province, Guangdong Province, and Hong Kong with a subtropical monsoon climate, Qinghai–Tibet Plateau with a plateau mountain climate, and Xinjiang with a temperate continental climate [24,25,26,27,28,29,30]. These findings suggest that children are exposed to low doses of antibiotics, and exposure to antibiotics is associated with adverse health effects in children. All the above reports are in temperate and subtropical regions. However, the tropical monsoon climate is warm and humid, which is more conducive to the incubation of vector microorganisms. Therefore, infectious diseases are more common in tropical regions, resulting in increased use of antibiotics. Thus, antibiotic resistance generally occurs in the tropical regions of the world [31,32]. Although antibiotic concentrations and antibiotic resistance genes have been reported in tropical waters, data on antibiotic exposure levels in children living in tropical monsoon climates are still limited [32,33,34].

Hainan Province is located in the Chinese tropical monsoon climate zone. It has a long coastline, numerous rivers, and developed water systems. It is one of the most advantageous areas in China for aquaculture. The increasing use of antibiotics in coastal aquaculture and animal husbandry in Hainan Province has led to the release of antibiotics into the surrounding aquatic environment, posing a certain risk to the fishery and aquatic environment of the province [35,36]. Although antibiotic concentration levels in waters have been reported in Hainan Province, there is still no report on antibiotic exposure levels in humans. Therefore, it is important to investigate antibiotic exposure levels in children in Hainan Province [37,38]. We hypothesized that school children in Hainan Province would demonstrate prevalent and detectable exposure to antibiotics internally.

In order to understand the level of antibiotic exposure in children in tropical areas, we conducted a small-scale study in Hainan Province, China. Pupils were recruited in primary schools in Hainan Province, and five commonly used antibiotics were considered, including fluoroquinolones, tetracyclines, sulfonamides, chloramphenicols, and macrolides. We evaluated the exposure level of antibiotics in children’s urinary antibiotics, and filled the data gap pertaining to human antibiotic exposure in tropical areas in China. In order to assess the health risks of antibiotic exposure in children, we carried out a risk assessment by calculating the hazard quotient (HQ) and hazard index (HI). At the same time, the urinary levels of 8-hydroxydeoxyguanosine (8-OHdG) and malondialdehyde (MDA) were assessed as biomarkers of DNA oxidative damage and lipid peroxidation, respectively, to explore the potential correlation between antibiotic exposure and oxidative stress injury.

## 2. Materials and Methods

### 2.1. Chemicals

In this study, sixteen antibiotics and three antibiotic metabolites were measured, grouped into five categories; namely, three chloramphenicol drugs [CAPs] (chloramphenicol [CAP], chlorophenicol [FF], thiamphenicol [TAP]), four macrolide drugs [MLs] (azithromycin [ATM], clarithromycin [CTM], roxithromycin [RTM], tylosin [TYL]), two tetracycline drugs [TCs] (oxytetracycline [OTC], 4-epoxytetracycline [4-epi-OTC]), four fluoroquinolone drugs [FQs] (ciprofloxacin [CIP], ofloxacin [OFX], enrofloxacin [EFX], norfloxacin [NFX]), and six sulfonamide drugs [SAs] (sulfamethazine [SM2], sulfamethoxazole [SMZ], sulfadiazine [SDZ], acetyl-sulfamethazine [AC-SM2] and acetyl-sulfamethoxazole [AC-SMZ] and trimethoprim [TMP]). In addition, nine isotope-labeled chemicals were selected as internal standards for quantitative analysis, including chloramphenicol-d_5_ (CAP-d_5_), florfenicol-d_5_ (FF-d_3_), thiamphenicol-d_5_ (TAP-d_5_), azithromycin-d_3_ (ATM-d_3_), erythromycin-^13^C-d_3_ (ETM-^13^C-d_3_), thibendazole-d_4_ (TBZ-d_4_), ciprofloxacin-d_8_ (CIP-d_8_), sulfadoxine-d_3_ (SDX-d_3_) and trimethoprim-d_3_ (TMP-d_3_). 8-OHdG and MDA standards were also purchased. All compounds were purchased from AccuStandard (New Haven, CT, USA) or Cambridge Isotope Laboratories (Andover, MA, USA).

Chromatographic-grade methanol, acetonitrile, ultrapure water, formic acid, citric acid, disodium hydrogen phosphate dodecahydrate, and disodium ethylenediaminetetraacetate were purchased from Fisher Scientifc, Waltham, MA, USA.

### 2.2. Study Population and Sample Collection

From November to December 2022, we recruited 304 students from six primary schools in four districts of Sanya City, Hainan Province. The primary-school students were all from grade one or two, aged between 6 and 9 years old. As a typical coastal tourist city, Sanya has a highly developed tourism industry and unique climatic conditions, which make it a representative case for studying regional antibiotic pollution exposure.

In the process of sample collection, the study strictly followed the standardized operation process. The first morning urine samples of each student were collected into a 15 mL polypropylene tube. After collection, the sample was quickly transported to the laboratory and transferred to an ultra-low temperature refrigerator at −20 °C for storage to minimize the risk of chemical degradation caused by temperature and light changes.

At the same time, in order to deeply understand the health status and living habits of the students, a questionnaire was designed. The questionnaire focuses on the individual demographic information, such as age, gender, height, birth weight, school, past medical history, sleep status, and dietary habits, filled out by their parents. During the survey, each participant was given a written informed consent form. They signed the consent voluntarily after clearly understanding all study details. This fully protected their right to be informed and make independent choices, ensuring the research was conducted ethically and smoothly. All activities in this study were approved by the Ethics Committee of Jinan University, China (JNUKY-2022-006).

### 2.3. Sample Preparation and Instrumental Analysis

Here, antibiotics were extracted from urine by solid-phase extraction using a previous method with slight modification [39]. A urine sample (1 mL) was added to internal standards and β-glucuronidase solution, and the pH was adjusted to 4 with Na_2_EDTA. The mixture was placed in a shaker and incubated overnight (37 °C). Then, the urine was extracted with a HLB column (Specifications: 3.9 × 150 mm, Manufacturer: Waters, Adsorbent Material: Oasist™ HLB 30 μm). The column was conditioned with 3 mL of methanol and 3 mL of ultrapure water in turn. After the urine was loaded, the impurities were removed with 6 mL of ultrapure water and 3 mL of 30% methanol water, and dried by vacuum suction. The antibiotics on the column were eluted with 6 mL mixed solvent (methanol and acetonitrile, *v*:*v* = 1:1). The extract was evaporated to almost dry under a mild nitrogen flow and dissolved in 0.5 mL of methanol. For the analysis of 8-OHdG and urine creatinine, urine samples were directly diluted with HPLC-grade water and internal standards (^15^N5-8-OHdG and d_5_-Creatinine) were added prior to instrumental analysis [40]. For the determination of malondialdehyde, 250 μL of Milli-Q water, 50 μL of urine, 50 μL of internal standard solution, and 150 μL of derivative agent (2,4-dinitrophenylhydrazine [DNPH]) were added to the injection bottle. These were then vortexed, and the mixture was placed in a 37 °C constant-temperature incubator for dark derivatization reaction for 80 min, waiting for instrumental determination.

The separation and quantification of all target compounds were conducted using an HPLC-MS/MS system (AB-Sciex 5600 triple quadrupole mass equipped with a Shimadzu Nexera-XZ LC system). For the analysis of antibiotics, chromatographic separation was performed on an Eclipse C_18_ column (4.6 mm × 100 mm, 5.0 μm; Agilent, Waltham, MA, USA). The mass spectrometry system used positive- and negative-ion spray ionization to quantify the target in the multi-reaction monitoring acquisition mode. The specific instrument method for antibiotics and 8-OHdG and urinary creatinine was similar to that used in our previous studies [39,40]. In addition, the specific instrument method for MDA is shown in the Supporting Information (SI, Table S1).

### 2.4. Quality Assurance and Quality Control (QA/QC)

In each batch of experiments, a urine sample was randomly selected as the matrix spiked sample, and the blank sample was water. Two blank samples, two blank spiked samples, two matrix spiked samples, and urine samples were analyzed in each batch of experiments to verify the feasibility of the method and monitor the potential matrix effect and accuracy of the experimental process. The internal standard recovery of antibiotics was 38 ± 17% to 147 ± 25%, and the recovery of 16 antibiotics and 3 metabolites in spiked samples was 78 ± 21% to 140 ± 20%. The limit of detection (LOD) of the instrument was calculated as the concentration of the analysis signal-to-noise ratio (S/N) equal to 3. The detailed QA/QC results are shown in Table S2.

### 2.5. Statistical Analysis

When calculating the mean, median, and detection frequency (DF) of the compounds, all concentrations below LOD were replaced by zero, and the concentration unit was ng/mL. Urinary creatinine correction data was only used for risk exposure assessment. All statistical analyses were performed using SPSS 26.0. In these analyses, the concentration of the target below the detection limit is represented by LOD divided by the root number 2. Due to the abnormal distribution of the antibiotic concentration, chi-square test was used to evaluate the relationship between socio-demographic characteristics and the frequency of antibiotic detection. Subsequently, binary logistic regression analysis was used to study the relationship between the detection rate of antibiotics and the frequency of dietary habits. All regressions included age, gender, BMI, parental education, and family income as covariates. When the OR value was greater than 1.0, the dietary habit was considered a risk factor. In order to verify the effect of antibiotic exposure on the two oxidative damage of lipid oxidation and DNA damage, the concentration of each antibiotic was calculated into quartiles (Q1–Q4). The multiple linear regression analysis was used to test the potential relationship between the two variables. The significance level of statistical analysis was set at *p* < 0.05.

### 2.6. Daily Exposure Estimation and Health Risk Assessment

Based on their urinary concentrations, the estimated daily intake (EDI) of an antibiotic can be estimated as follows [28]:

(1) $$EDI\left(\mu g/kg/day\right)=\frac{C_{a}\left(ng/mL\right)\times O_{cr}\left(mmol/day\right)}{C_{cr}\left(mmol/ml\right)\times W(kg)\times P\times 1000}$$

(2) $$EDI\left(\mu g/kg/day\right)=\frac{C_{a}\left(ng/mL\right)\times O_{cr}\left(mmol/day\right)}{C_{cr}(mmol/ml)\times W(kg)\times P\times 1000}\times \frac{MW_{P}}{MW_{M}}$$

The above Equation (1) was used for 16 parent antibiotics, and Equation (2) was used for three metabolites. C_a_ is the concentration of antibiotics in urine, O_cr_ is the daily output of creatinine in urine, and C_cr_ is the concentration of creatinine in urine. W is body weight. p is the percentage of antibiotic excretion in urine that remained unchanged as a glucuronide-binding substance [39] (Table S3). MW_P_ is the molecular weight of the parent compound, and MW_M_ is that of the metabolite. The daily output of creatinine in urine of healthy children aged 3–18 years is a height-based reference value [41]. Results from this simplified model may vary due to pharmacokinetic traits and population heterogeneity. Therefore, they should be treated as preliminary or suggestive of trends [42].

In addition, the health risk is also assessed by HQ and HI. HQ is the ratio of EDI to acceptable daily intake (ADI), where ADI is based on the endpoint of microbiological and toxicological effects. The ADI values used in this study are shown in Table S3 [39]. HI is the arithmetic sum of HQ, indicating the cumulative health risk of combined exposure to antibiotics. When HQ > 1 or HI > 1, there is a potential health risk.

## 3. Results and Discussion

### 3.1. Detection Frequencies and Concentrations of Antibiotics

**Figure 1:**
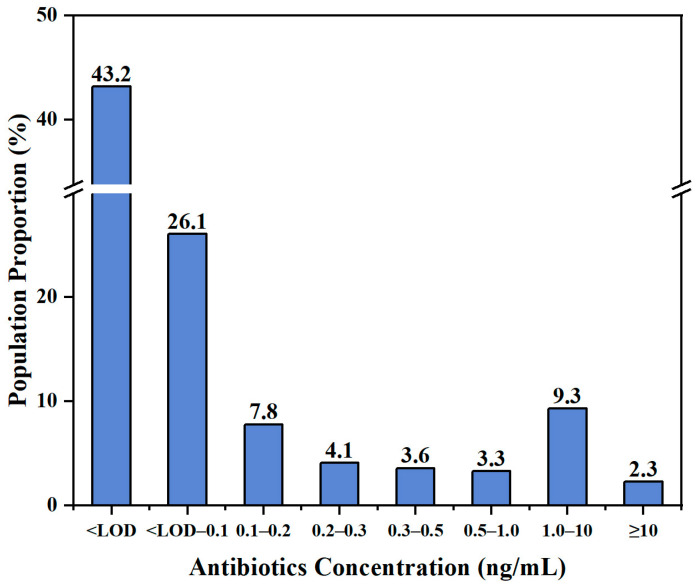
Frequency distribution of total concentrations of 19 antibiotics at different exposure levels (*n*= 302).

Among the five antibiotic classes, the DFs were 90%, 78.1%, 92.7%, 99.3%, and 100% for MLs, TCs FQs, SAs, and CAPs, respectively. The DFs of individual antibiotic ranged from 6.3% for OFX to 98.7% for FF. Four antibiotics were found in over 80% samples, including NFX, CAP, FF, and AC-SMZ (Table 1 and Figure 1). Currently, antibiotics in China are administratively categorized into three groups based on their primary authorized use. Human Antibiotics (HAs) include CAP, ATM, CTM, and RTM. Veterinary Antibiotics (VAs) include FF, TAP, TYL, OTC, 4-epi-OTC, and EFX. Antibiotics shared by humans and animals (H/VAs) include CIP, OFX, NFX, SM2, SMZ, SDZ, TMP, AC-SM2, and AC-SMZ. Due to the highly developed aquaculture industry in Hainan Province, the occurrence may indicate that the antibiotic levels in children in Sanya City is affected by the local aquaculture industry. Studies have shown that only 20–30% of antibiotics added to aquaculture water are absorbed by fish, and most antibiotics are input into the water body, even entering the human body in the form of drinking water [43]. In addition, a study on aquaculture in Hainan Province found that Sanya City was highly polluted by FQs and SAs [35]. This may be related to the frequent use of FQs as the main antibiotics in aquaculture and the relatively high stability of FQs in the aquatic environment. Sulfonamides are the most widely used and most common veterinary drugs in the world [44,45,46,47]. It is worth noting that CAPs are prohibited in food animal breeding in China [48], but their DFs reached 100% in children’s urine, although with low concentration levels.

The total concentration of all antibiotics in children’s urine was <LOD—4.58 × 10^3^ ng/mL. The median concentrations of MLs, TCs, FQs, SAs, and CAPs were <LOD, 0.8, <LOD, 0.02 and 0.16 ng/mL, with corresponding average concentration proportions of 4.36%, 84.1%, 9.31%, 0.779%, and 1.46%, respectively. Compared to several other studies in China, the occurrence of antibiotics in children in Hainan was different. For example, the median concentrations of TCs (0.8 ng/mL) and CAPs (0.16 ng/mL) in our study were higher than those in a cross-sectional survey in Shandong and Guangdong Provinces [25], which were (0.43 ng/mL) and (0.09 ng/mL), respectively (Table S4). In another study in Jiangsu Province, China, the median concentrations for MLs, TCs, FQs, SAs, and CAPs were 1.1, 0.8, 1.0, 2.1, and 2.7 ng/mL, respectively [49], most of them higher than our values. In Zhejiang Province, the median value of SAs (4.34 ng/mL) was higher than that in our study, but levels of MLs, FQs, and CAPs were lower than the detection limit [50]. Also, in Shanghai [28,51,52], Hong Kong [30], Qinghai–Tibet Plateau [27], and Xinjiang (adult) [53], the median concentration of antibiotics was lower than the detection limit. Those results all indicated that children in some southern coastal cities were facing exposure to higher antibiotic levels, compared with other cities in China.

In addition, current studies in China are distributed in different climate zones: Shandong falls within the temperate monsoon climate zone, while Jiangsu, Zhejiang, Shanghai, and Hong Kong are located in the subtropical monsoon climate zone. The Qinghai–Tibet Plateau features a highland mountain climate and Xinjiang has a temperate continental climate. From the above comparison of antibiotics in children, it can be concluded that environmental antibiotic pollution in tropical regions remains at low-to-moderate levels, significantly lower than in human activity-intensive subtropical areas, but higher than in plateau and arid regions. Although tropical conditions may promote antibiotic usage, their natural purification capacity, such as intense photolysis, efficient hydrolysis, microbial degradation, and precipitation dilution, likely effectively mitigates environmental exposure pressure.

### 3.2. Distribution of Antibiotics Among Selected Demographic Characteristics

The antibiotic distributions in children were compared according to different socio-demographic characteristics, including gender, BMI, parental education level, and household income (Table S5). The BMI was divided into three groups: the light-weight group (LG) (<18.5 kg/m^2^), normal-weight group (NG) (18.5–23.9 kg/m^2^), and overweight group (OG) (>23.9 kg/m^2^) [54].

As shown in Table S5, the study cohort comprised 204 boys (67.5%) and 98 girls (32.5%); no gender differences were observed. When considering BMI, the DF of OTC in the light-weight group was higher than other groups (NG: 42.7%, LG: 11.6%, and OG: 6.3%,) and such a difference was not observed for the DFs of AC-SMZ (NG: 77.4%, LG: 85.7%, and OG: 40%). With the improvement in the mother’s education level, the DFs of SMZ showed a downward trend (≤9 ys: 39.5%, 9–12 ys: 28.6%, and >12 ys: 24.6%). The DF of EFX was higher in households with moderate income (10,000–30,000: 28.6%), and the DF of OTC and AC-SMZ decreased with the increase in household income.

Although studies have shown that antibiotic use is associated with overweight in children [29,55], this correlation is not observed in our study and the DFs of OTC, and AC-SMZ were even higher in the light- or normal-weight group. In addition, families with low education levels may lack knowledge regarding medication, alongside other reasons, resulting in parents with low education levels being more inclined towards irrational views of antibiotics. At the same time, economic pressures often force people to live in poor environments, which are more likely to breed bacteria and viruses, thereby increasing the risk of infection. In the choice of medication, restricted by time, distance, and other conditions, pharmacies are the first choice for patients experiencing mild symptoms. They are more inclined to choose drugs including antibiotics with low prices but strong efficacy [56]. The antibiotic exposure rate of children in middle- and low-income areas is generally high, which is consistent with the trends in this study [57,58].

### 3.3. Dietary Habits and Antibiotic Detection

After adjusting for confounding variables, binary logistic regression analysis was used to explore the relationship between the DFs of antibiotics and dietary habits. The dietary habits included the weekly eating frequencies of vegetables, fruits, fried food, animal colostrums, protein powder, milk and dairy products, red meat, poultry meat, and aquatic products. According to the questionnaire, the frequency of different eating habits was divided into the following groups: low-frequency group (0 times/week), medium-frequency group (1–3 times/week), and high-frequency group (more than 4 times/week).

**Figure 2:**
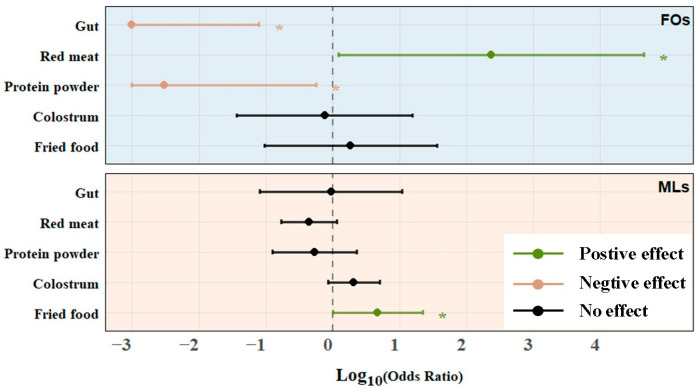
Binary logistic regression analysis of different dietary habits and antibiotic detection rate (*: *p* < 0.05).

As shown in Figure 2, positive correlations were found between high antibiotic DFs and the frequency of dietary intakes for MLs with fried foods (OR = 4.79, 95% CI 1.01–22.2), FQs with red meat (OR = 76.4, 95% CI 1.68–3479), CIP with aquatic products intake (OR = 3.96, 95% CI 1.32–11.9), and NFX with poultry meat intake (OR = 6.56, 95% CI 1.08–39.9). Also, several negative relationships were found for FQs with protein powder (OR = 0.003, 95% CI 0–0.567), viscera (OR = 0.0, 95% CI 0–0.08), and OFX with fruit intake (OR = 0.001, 95% CI 0–0.524). There was no significant correlation between the DFs of CAPs, TCs, and SAs and the frequency of any dietary habits in all analyses.

The statistical correlation between different types of antibiotics and the frequency of dietary habits was mainly concentrated in animal-derived foods. Fluoroquinolones are widely used in poultry, pig, and cattle farms to treat their colibacillosis. This may be the reason why the intake of red meat foods will increase the detection rate of FQ antibiotics [59,60]. One survey indicated that the three most commonly used fluoroquinolones in Chinese farms were ciprofloxacin, enrofloxacin, and norfloxacin, which also explained why the detection rate of norfloxacin increased significantly when poultry meat intake was high [61].

In addition, FQs, especially EFX, are antibiotics commonly used in aquaculture to treat diseases and promote growth [62,63]. For aquaculture, in order to effectively prevent and treat diseases in fish and shrimp, antibiotics with better stability in water are generally used. Although ciprofloxacin is not as widely used as enrofloxacin in aquaculture, the correlation in this study also gives us a warning. In particular, OR > 1 may indicate that high-frequency intake of red meat and aquatic products is associated with a higher risk of children’s exposure to FQs, which also reminds the relevant regulatory authorities to pay attention.

High intake of fried foods is positively correlated with the detection rate of MLs. This may be because people who frequently consume fried foods are more likely to be exposed to livestock and poultry meats with ML residues. Additionally, due to the thermostable properties of antibiotics, high-temperature frying cannot completely degrade them. In addition, the higher intake frequency of protein powder was significantly correlated with the decrease in FQ residue levels (*p* < 0.05). This phenomenon may be related to the physical and chemical effects of milk components: milk proteins such as casein can capture antibiotic molecules by hydrophobic binding or molecular chelation, reducing their intestinal absorption and bioavailability [64]. Furthermore, we found that fruit intake reduced the DF of OFX. Fruits are rich in dietary fiber. Studies have shown that a high fiber diet is positively correlated with the diversity of intestinal flora, and the diversity of flora improves the individual’s ability to metabolize antibiotics [65]. At the same time, fruits are rich in vitamins. For example, vitamin C is a highly effective antioxidant that reduces oxidative stress in the body and enhances the metabolic clearance of antibiotics [66].

### 3.4. Potential Health Risk Assessment of Antibiotic Exposure

**Figure 3:**
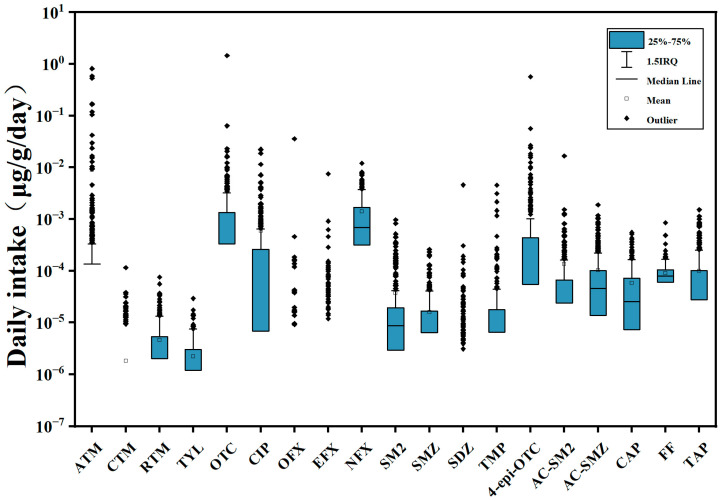
Daily intake of 16 antibiotics and 3 metabolites for children in Sanya, Hainan Province, China (*n* = 302).

As shown in Figure 3, the EDI of all antibiotics ranged from 0 to 1.45 μg/kg/day (OTC), with an overall average of 0.001 μg/kg/day. The majority of antibiotics (>99%) had calculated EDIs between 0.001 and 1.0 μg/kg/day.

In our study, 36% of children had a total EDI of >0.004 μg/kg/day for 19 targets, which was higher than the level of children in Hong Kong (8.4% of children had a total EDI > 0.002 μg/kg/day for 13 antibiotics in urine) [30]. Although direct numerical comparisons are limited by methodological differences, this suggests that exposure levels are higher in our cohort than in Hong Kong. Only 0.66% of children in our study had a total EDI of all antibiotics exceeding 1.0 μg/kg/day, which was much lower than 13.3% of children in Shanghai [51] and 15.2% of the elderly in Wanxi community [67].

**Figure 4:**
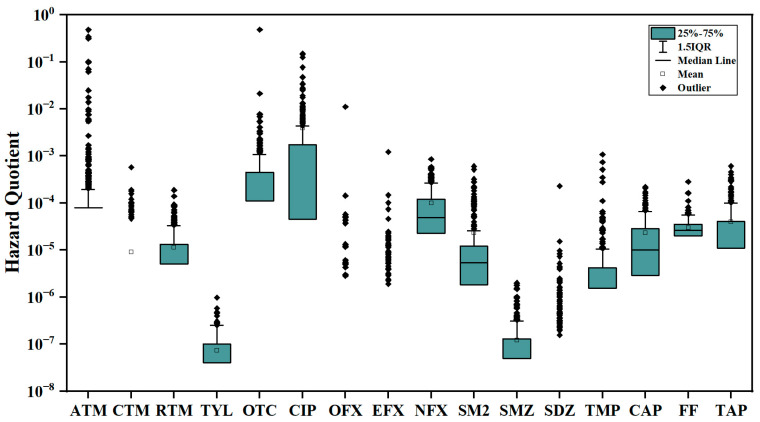
Hazard quotient (HQ) of 16 antibiotics for children in Sanya, Hainan Province, China (*n* = 302).

The hazard index is used to assess the impact of a single antibiotic exposure level on human health. The hazard quotient is used to assess the combined risk of co-exposure to multiple antibiotics. As shown in Figure 4, the HQ levels of all targets were below 1.0, indicating a lower health risk for children exposed to antibiotics. The HIs (HQ sum of SMZ, SM2, and SDZ) based on the endpoint of toxic effect was 0.007 (MeanMedian: 1.62 × 10^−5^ ± 0.012.77 × 10^−7^). The HIs (other 13 antibiotics) based on the endpoint of microbial effect was 3.50 (MeanMedian: 8.94 × 10^−4^ ± 0.00039.17 × 10^−8^). Among them, ATM, OTC, and CIP contributed the most, accounting for 45.6%, 18.1%, and 33.9%, respectively. These three antibiotics (ATM, OTC, and CIP) have been studied for common bacteria in the human intestine [68]. Laboratory studies have shown that antibiotics can affect the occurrence and development of immune and metabolic diseases by destroying the intestinal microbiota [69,70]. It is worth noting that due to the generally long-term stable dietary habits of the population, this persistent low-dose antibiotic exposure may have a cumulative impact on the composition of gut microbiota, thereby potentially increasing health risks [71]. Low levels of antibiotics can enrich resistant mutants in a population, even at concentrations several hundred-fold below the minimum inhibitory concentration, highlighting their role in driving resistance evolution [72,73]. Therefore, long-term exposure to low-dose antibiotics may have significant implications that cannot be ignored.

### 3.5. Lipid Peroxidation and DNA Damage by Antibiotics

Lipids are the structural basis of cell membranes, especially unsaturated fatty acids [74]. Antibiotics may cause cell damage through mechanisms such as inducing lipid peroxidation, destroying membrane integrity, and generating reactive aldehydes, thereby promoting the occurrence and development of various diseases, such as cardiovascular diseases and liver diseases [75,76]. Malondialdehyde (MDA) is a biomarker of lipid peroxidation [77]. In addition, we also measured the concentration of 8-OHdG in children [78]. 8-OHdG is the product of guanine base oxidation in the DNA chain, which is closely related to gene mutation, apoptosis, and cancer. The increase in the 8-OHdG level indicates that the stability of genome is impaired, which may promote the development of disease [79]. We explored the relationships between children urinary antibiotics and the levels of MDA or 8-OHdG to partly reflect the potential health risks forming antibiotic exposure.

In the present study, the mean value of MDA concentration in all urine samples was 163 ± 9.07 ng/mL. The concentration of the first quartile of MDA was used as a reference in the multiple linear regression analysis. MLs and CAPs at Q4 showed significant positive correlations with MDA levels. SAs at Q3-Q4 were positively correlated with MDA. TCs demonstrated no significant effects across all concentrations, while FQs reached significance only at Q2 (Figure S1). The pro-oxidative effects of MLs and CAPs align with their mechanism of disrupting cell-membrane lipid homeostasis. Similarly, the dose–response relationship of SAs is consistent with findings in fish models, where SAs significantly increased MDA levels [80,81]. Notably, the limited significance observed for FQs suggests potential inter-individual metabolic variations, contrasting with their established role as core drivers of oxidative damage in animal models and indicating possible species-specific responses [82].

**Figure 5:**
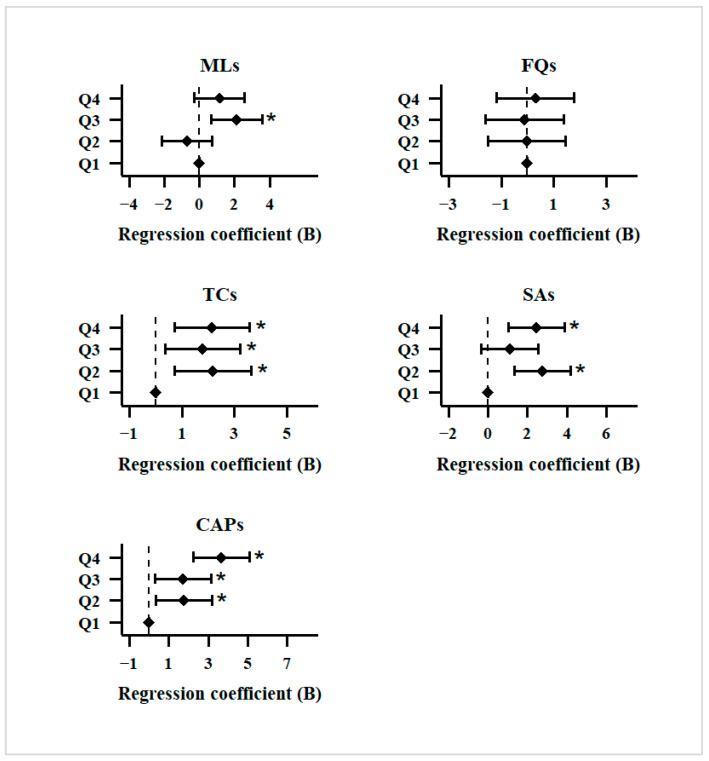
Multiple linear regression analysis of five classes of antibiotics and 8-OHdG (*: *p* < 0.05).

For DNA oxidative damage biomarker 8-OHdG, the mean value of 8-OHdG concentration in all urine samples was 8.12 ± 0.26 ng/mL. All antibiotic classes except FQs demonstrated significant positive correlations with 8-OHdG concentrations at higher quantiles. Specifically, TCs and CAPs exhibited consistent positive associations from Q2 to Q4, with 8-OHdG levels increasing by 32% (TCs) and 58% (CAPs), respectively, when compared to the Q1 reference group (Figure 5 and Figure S3). The dose-dependent correlations suggest potential genotoxic effects at elevated concentrations. This is particularly evident for CAPs, which showed a 58% increase in 8-OHdG levels, similar to a finding in the Shanghai study [83]. Existing studies have reported that CAPs can generate sustained reactive oxygen species (ROS) through redox cycling, leading to DNA base oxidation, with the presence of copper ions amplifying this damaging effect [84]. Research has shown that tetracyclines induce oxidative stress in zebrafish larvae [85], and macrolides can promote malignant progression by inhibiting autophagy and inducing ROS accumulation [86]. However, other studies indicate that certain bacteriostatic antibiotics, such as tetracyclines, may not directly trigger ROS production [87].

## 4. Conclusions

Children in Sanya City, Hainan Province, China, a tropical monsoon climate zone in China, are widely exposed to antibiotics. Tropical regions’ self-purification capacity may effectively offset antibiotic usage, maintaining lower contamination levels than subtropical areas. Regular consumption of poultry meat, red meat, and aquatic products may be related to the intake of more antibiotics. Protein powder and fruit intake may exert a protective effect. Although the EDI of most antibiotics is less than 1.0 μg/kg/day, the cumulative risk based on microbial endpoints also reminds us of the risk of long-term exposure at low doses. In addition, the close correlation between DNA damage and lipid peroxidation markers with antibiotics also indicates potential health risks from exposure.

This study investigated the antibiotic exposure in children in Sanya City through biological monitoring methods, filling the gap in relevant data. However, this study also has some limitations. In the process of data collection, the diet habit questionnaire relies on the subjective memory of the research object or its guardian, which may have memory bias and affect the accuracy of the data. At the same time, the research only focuses on some dietary factors, and does not fully incorporate potential influencing factors such as family medication history and personal hygiene habits. It is difficult to fully analyze the complex causes of antibiotic exposure. Future research can further expand and improve in this direction.

## Figures and Tables

**Table 1:** Detection frequencies and concentrations (ng/mL) of antibiotics in the urine of children (*n*= 302).

Antibiotics	Abbreviation	Category	*n*	Percentiles			Mean	DF (%)
				50th	75th	95th		
**Macrolide**	**MLs ^a^**		272	nd	0.014	0.12	0.81 ± 0.54	90.0
Azithromycin	ATM	HAs	82	nd	0.037	3.18	3.24 ± 2.17	27.2
Clarithromycin	CTM	HAs	166	nd	0.097	0.028	0.007 ± 0.0006	55.0
Roxithromycin	RTM	HAs	165	nd	0.02	0.07	0.02 ± 0.002	54.6
Tylosin	TYL	VAs	215	0.005	0.013	0.03	0.009 ± 0.0009	71.2
**Tetracycline**	**TCs**		236	0.8	3.45	25.8	15.5 ± 10.8	78.1
Oxytetracycline	OTC	VAs	168	1.16	4.94	22.2	20.2 ± 15.2	55.6
4-epi- oxytetracycline	4-epi-OTC	VAs	190	0.24	1.64	25.1	10.8 ± 6.49	62.9
**Fluoroquinolone**	**FQs**		280	nd	1.05	9.32	1.72 ± 0.34	92.7
Ciprofloxacin	CIP	H/VAs	154	nd	0.82	6.75	1.79 ± 0.59	50.7
Ofloxacin	OFX	H/VAs	19	nd	nd	0.07	0.54 ± 0.75	6.3
Enrofloxacin	EFX	VAs	51	nd	nd	0.11	0.05 ± 0.04	16.9
Norfloxacin	NFX	H/VAs	252	2.18	5.22	17.76	4.56 ± 0.42	83.4
**Sulfonamide**	**SAs**		300	0.02	0.071	0.47	0.14 ± 0.02	99.3
Sulfamethazine	SM2	H/VAs	231	0.03	0.07	0.6	0.12 ± 0.02	76.5
Sulfamethoxazole	SMZ	H/VAs	186	0.001	0.02	0.11	0.02 ± 0.003	61.9
Sulfadiazine	SDZ	H/VAs	75	nd	nd	0.08	0.06 ± 0.06	24.2
Trimethoprim	TMP	H/VAs	205	0.02	0.06	0.41	0.2 ± 0.08	67.9
Acetyl- sulfamethazine	AC-SM2	H/VAs	220	0.05	0.14	0.92	0.3 ± 0.03	72.8
Acetyl- sulfamethoxazole	AC-SMZ	H/VAs	269	0.08	0.16	0.71	0.17 ± 0.03	89.1
**Chloramphenicol**	**CAPs**		302	0.16	0.29	0.85	0.27 ± 0.02	100
Chloramphenicol	CAP	HAs	254	0.13	0.36	1.02	0.29 ± 0.03	84.1
Florfenicol	FF	VAs	298	0.2	0.25	0.41	0.23 ± 0.01	98.7
Thiamphenicol	TAP	VAs	186	nd	0.33	1.37	0.3 ± 0.28	61.6

Notes: nd: <limits of detection (LODs). a: Sum of concentrations of antibiotics in the corresponding category for the individual.

## Data Availability

The raw data supporting the conclusions of this article will be made available by the authors on request.

## Supplementary Materials

The following supporting information can be downloaded at: https://www.mdpi.com/article/10.3390/toxics13121089/s1, Figure S1: Multiple linear regression analysis of five classes of antibiotics and MDA; Figure S2: Relationships between higher concentrations of antibiotic and 8-OHdG; Table S1: Optimized parameters of mass spectrometry for the quantification of MDA; Table S2: The results of QA/QC during sample preparation and quantification; Table S3: Excretion proportions of 19 antibiotics in urine as free and glucuronide-conjugated forms, and acceptable daily intakes (ADIs) of antibiotics based on different effect endpoints; Table S4: Median antibiotic concentrations (ng/mL) in urine samples from populations across different monsoon climate zones; Table S5: Detection rates of urinary antibiotics in different demographic characteristics among students in Hainan, China.
