# Supplementary material for: Antibiotic Exposure in School Children in Tropical Environments: Impact of Dietary Habits and Potential Health Risks
Source: Toxics. 2025 Dec 18;13(12):1089. doi: 10.3390/toxics13121089 (PMC12737428; doi:10.3390/toxics13121089)
Supplement: Supplementary file 1 [file toxics-13-01089-s001.zip › toxics-4010426-supplementary.pdf]

Supporting Information

For

Antibiotic Exposure in School Children in Tropical Environments: Impact of Dietary Habits and Potential Health Risks

Lin Zhao [1], Xin-Yu Wang [1], Yang Xiang [1], Ting-Ting Xu [1], Shi-Jian Liu [2,*] and Xiao-Ya Lin [1,3] and Ying Guo [1,*]

[1] Guangdong Key Laboratory of Environmental Pollution and Health, School of Environment and Climate, Jinan University, Guangzhou 511443, China; zl1013@stu.jnu.edu.cn (L.Z.); xinyuwang@stu2023.jnu.edu.cn (X.-Y.W.); 880216bkpp@stu2023.jnu.edu.cn (Y.X.); xutingting@jnu.edu.cn (T.-T.X.); linxiaoya@stu2025.jnu.edu.cn (X.-Y.L.)
[2] Clinical Research Unit, Shanghai Municipal Hospital of Traditional Chinese Medicine, Shanghai University of Traditional Chinese Medicine, Shanghai 201203, China
[3] Hainan Branch, Shanghai Children's Medical Center, School of Medicine, Shanghai Jiao Tong University, Sanya 572000, China
* Correspondence: shijianliu@dhdmu.edu.cn (S.-J.L.); yingguo2004@jnu.edu.cn (Y.G.)

NO. OF FIGURES: 2

NO. OF TABLES: 5

NO. OF PAGES: 10

Malondialdehyde in urine

First, prepare the derivative agent: 0.0198g 2,4-dinitrophenylhydrazine (DNPH) was dissolved in 10 mL acetonitrile (containing 2 % acetic acid) and diluted to 1 mmol / L with 2 % formic acid aqueous solution. Then, 250μL of Milli-Q water, 50μL of urine, 50μL of internal standard solution, and 150μL of derivative agent (DNPH) were added to the injection bottle. These were vortexed for mixing, then placed in a 37°C constant-temperature incubator for dark derivatization reaction for 80 minutes, waiting instrumental determination. The specific instrument method is shown in Table S1.

Table S1. Optimized parameters of mass spectrometry for the quantification of MDA.

Chemicals	Q1	Q3	DP	EP	CE	CXP	CUR	CAD	GS1	GS2
MDA-DNPH	235	159	30	10	30	8.0	20	5	50	50
d_2-MDA-DNPH	237	161	30	10	30	8.0	20	5	50	50

Table S2. The results of QA/QC during sample preparation and quantification (mean±SD).

Antibiotics	Abbreviation	LOD (ng/mL)	LOQ (ng/mL)	Procedural blank (n=12)	Blank spike (n=12)	Sample spike (n=12)	Samples (n=302)
Azithromycin	ATM	0.038	0.11	-[a]	118%±23%	119%±21%	-
Clarithromycin	CTM	0.004	0.011	-	129%±23%	128%±21%	-
Roxithromycin	RTM	0.01	0.03	-	83%±10%	110%±18%	-
Tylosin	TYL	0.004	0.012	-	78%±21%	93%±23%	
Oxytetracycline	OTC	1.95	2.5	-	125%±20%	140%±20%	-
4-epi-oxytetracycline	4-epi-OTC	0.12	0.41	-	124%±30%	158%±35%	-
Ciprofloxacin	CIP	0.024	0.072	-	85%±30%	78%±18%	-
Ofloxacin	OFX	0.060	0.179	-	93%±32%	92%±21%	-
Enrofloxacin	EFX	0.037	0.109	-	82%±36%	74%±18%	-
Norfloxacin	NFX	0.865	2.59	-	99%±16%	100%±28%	-
Sulfamethazine	SM2	0.017	0.05	-	100%±7.0%	94%±18%	-
Sulfamethoxazole	SMZ	0.009	0.026	-	106%±8.0%	89%±19%	-
Sulfadiazine	SDZ	0.023	0.068	-	83%±20%	108%±23%	
Acetyl-sulfamethazine	AC-SM2	0.012	0.037	-	114%±11%	124%±24%	-
Acetyl-sulfamethoxazole	AC-SMZ	0.007	0.022	-	92%±23%	83%±23%	-
Trimethoprim	TMP	0.014	0.04	-	114%±13%	107%±17%	-
Chloramphenicol	CAP	0.039	0.11	-	105%±15%	86%±19%	
Florfenicol	FF	0.099	0.29	-	117%±17%	97%±14%	-
Thiamphenicol	TAP	0.095	0.28	-	110%±17%	111%±25%	-
Azithromycin-d_3	ATM-d_3	-		55%±25%	38%±17%	143%±19%	147%±25%
Erythromycin-^{13}C-d_3	ETM-^{13}C-d_3	-		72%±28%	69%±20%	65%±21%	74%±74%
Thiabendazole-d_4	TBZ-d_4	-		95%±7.8%	97%±8.6%	84%±13%	76%±14%
Ciprofloxacin-d_8	CIP-d_8	-		60%±20%	62%±16%	126%±25%	127%±18%
Sulfadoxine-d_3	SDX-d_3	-		78%±10%	80%±7%	78%±11%	70%±16%
Trimethoprim-d_3	TMP-d_3	-		94%±10%	84%±12%	92%±19%	90%±11%
Chloramphenicol-d_5	CAP-d_5	-		93%±22%	94%±15%	82%±22%	96%±28%
Florfenicol-d_5	FF-d_3	-		96%±8%	97%±19%	83%±18%	106%±20%
Thiamphenicol-d_5	TAP-d_3	-		95%±10%	97%±17%	83%±18%	84%±22%

a.no available

Table S3. Excretion proportions of 19 antibiotics in urine as free and glucuronide-conjugated forms, and acceptable daily intakes (ADIs) of antibiotics based on different effect endpoints.

Antibiotics	Abbreviation	Excretion rate via urine (%)			ADI (μg/kg-bw/day)	Effect endpoint
		Free	Glucuronid	Adopted		
**Phenicols**						
Chloramphenicol	CAP	10.1(8.0-15.0)	83.3	93.4	2.5	Microbiological
Florfenicol[a]	FF	47.5	-	47.5	3	Microbiological
Thiamphenicol	TAP	55.1(41.1-70.2)	0.5(0.1-1.0)	55.6	2.5	Microbiological
**Macrolides**						
Azithromycin	ATM	5.1(4.6-5.5)	-	5.1	1.7	Microbiological
Clarithromycin	CIM	33.7(14.4-60)	-	33.7	0.2	Microbiological
Roxithromycin	RTM	66.7(57-74.5)	-	66.7	0.4	Microbiological
Tylosin	TYL	78	-	78	30	Microbiological
**Tetracyclines**						
Oxytetracycline	OTC	66.7(50-80)	-	66.7	3	Microbiological
4-epi-oxytetracycline	A-epi-OTC	70	-	70	-	-
**Fluoroquinolones**						
Ciprofloxacin	CIP	53.8(29.5-83.7)	-	53.8	0.15	Microbiological
Ofloxacin	OFX	75.8(70-82)	-	75.8	3.2	Microbiological
Enrofloxacin[a]	EFX	21.0	-	21.0	6.2	Microbiological
Norfloxacin	NFX	61.5	-	61.5	14	Microbiological
**Sulfonamides**						
Sulfamethazine	SM2	65.9(41.7-77.5)		65.9	1.6	Toxicological
Sulfamethoxazole	SMZ	15.2(9.7-20)	12.3(9.6-15)	27.5	130	Toxicological
Sulfadiazine	SDZ	52.3(30.0-78.0)		52.3	20	Toxicological
Trimethoprim	TMP	60.5(48-80)		60.5	4.2	Microbiological
Acetyl-sulfamethazine	AC-SM2	45		45	-	-
Acetyl-Sulfamethoxazole	AC-SMZ	39		39		--

Notes: a: due to a lack of human pharmacokinetic data, the urinary excretion rates from pigs or chickens were used;

-, no excretion of antibiotics as glucuronide forms or no ADI were obtained.

Table S4. Median antibiotic concentrations (ng/mL) in urine samples from populations across different monsoon climate zones.

Monsoon climate	Province	N	Age	MLs	TCs	FQs	SAs	CAPs	Reference
Temperate, Subtropical	Shandong Guangdong	817	6-12	0.82	0.43	0.34	0.12	0.09	[1]
Subtropical	Jiangsu	295	3-7	0.8	1.0	2.7	2.7	1.1	[2]
	Zhejiang	297	7-12	<LOD	0.67	<LOD	4.34	<LOD	[3]
	Shanghai	684	7-11	<LOD	<LOD	<LOD	<LOD	<LOD	[4]
	Shanghai	284	8-11	<LOD	<LOD	<LOD	<LOD	<LOD	[5]
	Shanghai	2119	6-12	<LOD	<LOD	<LOD	<LOD	<LOD	[6]
	Hong Kong	31	3-6	<LOD	<LOD	<LOD	<LOD	<LOD	[7]
Highland mountain	Qinghai–Tibet Plateau	249	8-12	<LOD	<LOD	<LOD	<LOD	<LOD	[8]
Temperate continental	Xinjiang	873	35-75	<LOD	<LOD	<LOD	<LOD	<LOD	[9]
Tropical	Hainan	302	6-9	0.003	0.8	<LOD	0.02	0.16	This study

Table S5. Detection rates of urinary antibiotics in different demographic characteristics among students in Hainan, China (N=302).

	N	ATM	CTM	RTM	TYL	OTC	CIP	OFX	EFX	NFX
**Sex**	302									
Boys	204	58(28.4)	19(9.3)	55(27.0)	31(15.2)	118(39.1)	105(51.5)	19(9.3)	34(16.7)	186(91.2)
Girls	98	24(24.5)	9(9.2)	22(22.4)	14(14.3)	50(16.6)	46(46.9)	3(3.1)	16(16.3)	87(88.8)
**BMI (kg/m^2)**										
<18.5	243	65(26.7)	21(8.6)	58(23.9)	35(14.4)	**129(42.7)**	119(49.0)	18(7.4)	37(15.2)	222(91.4)
18.5-23.9	49	15(30.0)	7(14.3)	17(34.7)	9(18.4)	**35(11.6)**	28(57.1)	2(4.1)	12(24.5)	44(89.8)
>23.9	10	2(20.0)	0(0)	2(20.0)	1(10.0)	**4(1.3)**	4(40.0)	2(20.0)	1(10.0)	7(70.0)
**Father's education (year)**										
≤9	139	35(25.2)	17(12.2)	37(26.6)	24(17.3)	82(27.2)	68(48.9)	12(8.6)	16(11.5)	124(89.2)
9-12	35	12(34.3)	4(11.4)	8(22.9)	7(20.0)	20(6.6)	16(45.7)	5(14.3)	7(20.0)	29(82.9)
>12	128	35(27.3)	7(5.5)	2(25.0)	14(10.9)	66(21.9)	67(52.3)	5(3.9)	27(21.1)	120(93.8)
**Mother's education (year)**										
≤9	152	38(25.0)	18(11.8)	44(28.9)	25(16.4)	86(28.5)	76(50.0)	14(9.2)	24(15.8)	134(88.2)
9-12	28	11(39.3)	3(10.7)	6(21.4)	4(14.3)	19(6.3)	10(35.7)	1(3.6)	2(7.1)	27(96.4)
>12	122	33(27.0)	7(5.7)	27(22.1)	16(13.1)	63(20.9)	65(53.3)	7(5.7)	24(19.7)	112(91.8)
**Family income (yuan/ year)**										
<10000	182	49(26.9)	17(9.3)	46(25.3)	30(16.5)	**182(60.3)**	91(50.0)	16(8.8)	**27(14.8)**	163(89.6)
10000-30000	56	14(25.0)	7(12.5)	15(26.8)	6(10.7)	**56(18.5)**	33(58.9)	3(5.4)	**16(28.6)**	50(89.3)
>30000	5	0(0)	0(0)	1(20.0)	0(0)	**5(1.7)**	2(40.0)	0(0)	**0(0)**	4(80.0)
Not know	59	19(32.2)	4(6.8)	15(25.4)	9(15.3)	**59(19.5)**	25(42.4)	3(5.1)	**7(11.9)**	56(94.9)

Table S5. Detection rates of urinary antibiotics in different demographic characteristics among students in Hainan, China (n=302). (continued).

	N	SM2	SMZ	SDZ	TMP	4-epi-OTC	AC-SM2	AC-SMZ	CAP	FF	TAP
**Sex**	302										
Boys	204	130(63.7)	68(33.3)	39(19.1)	107(52.5)	129(42.7)	139(68.1)	160(78.4)	178(87.3)	203(99.5)	158(77.5)
Girls	98	58(59.2)	30(30.6)	16(16.3)	52(53.1)	61(20.2)	61(62.2)	74(75.5)	78(79.6)	98(100.0)	78(79.6)
**BMI (kg/m^2)**											
<18.5	243	147(60.5)	79(32.5)	45(18.5)	128(52.7)	149(49.3)	162(66.7)	**188(77.4)**	206(84.8)	242(99.6)	187(77.0)
18.5-23.9	49	35(71.4)	18(36.7)	7(14.3)	27(55.1)	33(10.9)	33(67.3)	**42(85.7)**	42(85.7)	49(100)	43(87.8)
>23.9	10	6(60.0)	1(10.0)	3(30.0)	4(40.0)	8(2.6)	5(50.0)	**4(40.0)**	8(80.0)	10(100)	6(60.0)
**Father's education (year)**											
≤9	139	91(65.5)	54(38.8)	28(20.1)	74(53.2)	91(30.1)	86(61.9)	113(81.3)	121(87.1)	139(100)	107(77.0)
9-12	35	19(54.3)	11(31.4)	10(28.6)	19(54.3)	17(5.6)	27(77.1)	26(74.3)	31(88.6)	35(100)	25(71.4)
>12	128	78(60.9)	33(25.8)	17(13.3)	66(51.6)	82(27.2)	87(68.0)	95(74.2)	104(81.3)	127(99.2)	104(81.3)
**Mother's education (year)**											
≤9	152	96(63.2)	**60(39.5)**	30(19.7)	86(56.6)	100(33.1)	98(64.5)	122(80.3)	129(84.9)	152(100)	119(78.3)
9-12	28	17(60.7)	**8(28.6)**	5(17.9)	14(50.0)	15(5)	18(64.3)	23(82.1)	25(89.3)	28(100)	22(78.6)
>12	122	75(61.5)	**30(24.6)**	20(16.4)	59(48.4)	75(24.8)	84(68.9)	89(73.0)	102(83.6)	121(99.2)	95(77.9)
**Family income (yuan/ year)**											
<10000	182	112(61.5)	69(37.9)	37(20.3)	100(54.9)	117(38.7)	118(64.8)	**152(83.5)**	155(85.2)	181(99.5)	142(78.0)
10000-30000	56	39(69.6)	19(33.9)	6(14.7)	25(44.6)	36(11.9)	35(62.5)	**42(75)**	44(78.6)	56(100)	46(82.1)
>30000	5	5(100)	2(40.0)	0(0)	2(40.0)	3(1)	3(60.0)	**3(60.0)**	5(100)	5(100)	5(100)
Not know	59	32(54.2)	8(13.6)	12(20.3)	32(54.2)	34(11.3)	44(74.6)	**37(62.7)**	52(88.1)	59(100)	43(72.9)

Bold: $p<0.05$

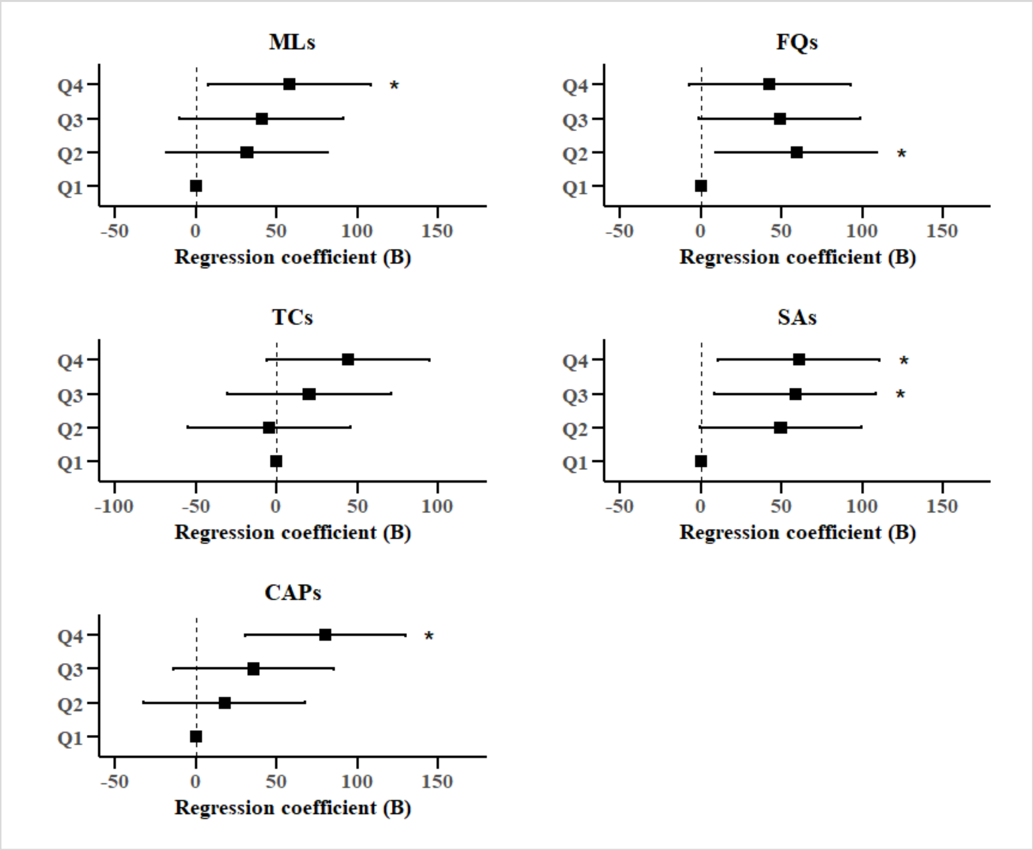

Figure S1

Multiple linear regression analysis of five classes of antibiotics and MDA (*: $p<0.05$).

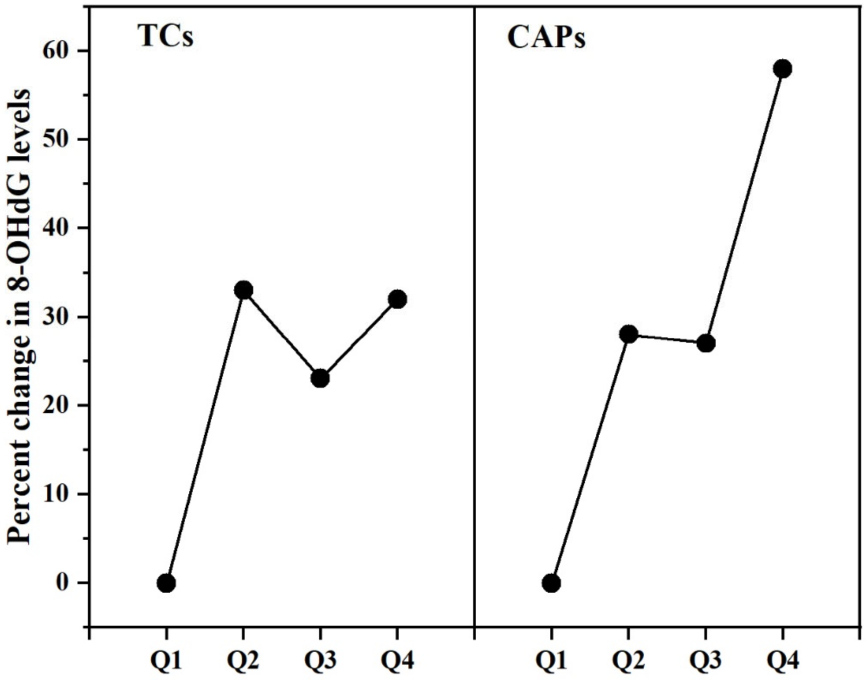

Figure S2

Relationships between higher concentrations of antibiotic and 8-OHdG

(Quartile 1, 2, 3 and 4 represent 0–25 %, 25–50 %, 50–75 % and 75–100 % of the data, respectively)

References:

1. Hu, Yabin, Juan Li, Tao Yuan, Tingting Yu, Yao Chen, Huijun Kong, Cuilan Lin, Zhemin Shen, Ying Tian, Shilu Tong, Xiaodan Yu, and Shijian Liu. "Exposure to Antibiotics and Precocious Puberty in Children: A School-Based Cross-Sectional Study in China." *Environmental Research* 212 (2022): 113365.
2. Wang, Xingchen, Huamin Sun, Yijing Zhou, Dongyu Zheng, Jianping Huang, Xiangping Liu, Xin Xu, Wenfang Sun, and Xiang Huo. " Urinary Antibiotic Level and Influencing Factors in Children Residing in Jiangsu Province." *Chinese Journal of Food Hygiene* 36, no. 03 (2024): 300-08.
3. Cheng, Xi. Urinary Antibiotics Exposure of Pregnant Women and School-Age Children and Health Risk Assessment of Antibiotic Ingestion in Seafood in Zhoushan City Bachelor's degree, 2021.
4. Wang, Hexing, Chuanxi Tang, Yuanping Wang, Minghui Han, Feng Jiang, Lufang Jiang, Jingui Wu, Chaowei Fu, Yue Chen, and Qingwu Jiang. "Urinary Antibiotic Level of School Children in Shanghai, East China, 2017–2020." *Environmental Pollution* 291 (2021): 118167.
5. Wang, Hexing, Chuanxi Tang, Jiaqi Yang, Na Wang, Feng Jiang, Qinghua Xia, Gengsheng He, Yue Chen, and Qingwu Jiang. "Predictors of Urinary Antibiotics in Children of Shanghai and Health Risk Assessment." *Environment International* 121 (2018): 507-14.
6. Zhang, Yu, Weifeng Tang, Yuqing Wang, Min Nian, Fan Jiang, Jun Zhang, and Qian Chen. "Environmental Antibiotics Exposure in School-Age Children in Shanghai and Health Risk Assessment: A Population-Based Representative Investigation." *Science of The Total Environment* 824 (2022): 153859.
7. Li, Na, Keith W. K. Ho, Guang-Guo Ying, and Wen-Jing Deng. "Veterinary Antibiotics in Food, Drinking Water, and the Urine of Preschool Children in Hong Kong." *Environment International* 108 (2017): 246-52.
8. Huang, Yushan, Zuhong Zhang, Tianchun Hou, Jingfang Shi, Wenjie Huang, Zhao Bai, Danfeng Long, Xiaodan Huang, and Shijuan Yan. "Antibiotic Burden of School Children from Tibetan, Hui, and Han Groups in the Qinghai–Tibetan Plateau." *PloS one* 15, no. 2 (2020): e0229205.
9. Chu, Lei, Hexing Wang, Deqi Su, Huanwen Zhang, Bahegu Yimingniyazi, Dilihumaer Aili, Tao Luo, Zewen Zhang, Jianghong Dai, and Qingwu Jiang. "Urinary Antibiotics and Dietary Determinants in Adults in Xinjiang, West China." *Nutrients* 14, no. 22 (2022): 4748.
